# Using a Taguchi DOE to investigate factors and interactions affecting germination in *Miscanthus sinensis*

**DOI:** 10.1038/s41598-020-58322-x

**Published:** 2020-01-31

**Authors:** Danny Awty-Carroll, Sreenivas Ravella, John Clifton-Brown, Paul Robson

**Affiliations:** Institute of Biological, Environmental and Rural Sciences, Gogerddan, University of Aberystwyth, Aberystwyth, SY23 3EE UK

**Keywords:** Light responses, Plant hormones, Plant physiology, Plant stress responses

## Abstract

The *Miscanthus* genus of perennial grasses is grown for bioenergy and biorenewable feedstocks. Most *Miscanthus* crop is *M* × *giganteus* which is rhizome propagated and therefore difficult to multiply at large scale. Seed-based propagation of new hybrids is being developed, but *Miscanthus* is difficult to establish from seed especially in the field. *Miscanthus* is often grown on marginal land adding to the challenge of successfully establishing the crop. Improved understanding of the limits and biology of germination in *Miscanthus* species is needed. Seed germination is affected by physical and chemical factors that impact germination differently depending on level of exposure. In this investigation of *Miscanthus* germination, four hormones plus water stress were investigated and the range over which these factors affect germination was determined. An efficient Taguchi experimental design was used to assess the five factors in combination with the effects of light and seed priming. This determined an example of a set of optimum conditions for *Miscanthus* germination and demonstrated how this could change based on fixing one condition. The experiment showed how environmental stress impacted germination and how treatments such as gibberellic acid could be used to mitigate stress.

## Introduction

*Miscanthus* is a genus of *Poaceae* that is being developed for biomass for biorenewable energy and bioproducts, as it is able to produce over 10 t DM ha^−1^ in a temperate climate^1^. The crop is currently of a single hybrid (*M* × *giganteus Greef et Deu* (Aksel Olson)^2^) and is mostly propagated through inefficient and expensive clonal propagation from rhizomes. An alternative seed-based propagation would be preferred to increase the scale of production of the crop that is needed to deliver sufficient global impact^3,4^. This genotype would be of *M. sinensis*, *M. sacchariflorus* or a interspecies hybrid, *Miscanthus* seeds are harvested from panicles^5^. Successful seed-based establishment is difficult^6^, particularly because it is proposed that *Miscanthus* can be planted on marginal land to avoid competing with food crops^7–9^. A synthetic population of *M. sinensis* can be used for studying germination due to the availability of seed^4,6,10^. Marginal land suffers from a range of possible issues including water and salinity stress that will further exacerbate the problems of seed propagation^11^. Thus, a broad understanding of the physical and hormonal interactions on Miscanthus seed germination and early growth is important to improve both seed-based multiplication and to identify novel treatments to increase crop establishment on marginal land.

The Taguchi method was first used in manufacturing^12^, and is now a widely adopted method for efficient experimental design^13^. It is designed to test multiple factors together by first defining the range of the factors and then defining the noise^14^. Orthogonal arrays (OA) are used to account for the noise using multi-variant statistical techniques^14^. The tabulated arrays allow a maximum number of effects to be compared orthogonally in an unbiased manor, using a minimal number of experiments^15^. This method uses analysis of variance (ANOVA) to identify which variables in a group are contributing to the variation^16,17^. This makes it useful for processes such as germination with many variables, although Tong, Su, & Wang^14^ suggest that it is less suited to studies where the variables react interdependently with each other. The Taguchi method has not been widely adopted in the biological sciences^15^; however, Yaldagard, Mortazavi, & Tabatabaie^13^ employed the Taguchi method to identify factors affecting germination in barley, which suggests that it would be suitable for studying the complex interactions that affect germination in *Miscanthus*. Abscisic acid, gibberellic acid, brassinosteroid, and auxin have previously been shown to have a direct effect on seed germination or development^18–21^. These hormones may be used to easily treat seed in the aqueous form (e.g. excluding ethylene gas) allowing a consistent delivery of treatments for direct comparisons. *Miscanthus* is often grown on marginal land and therefore the crop needs to establish in poor conditions^3,22^. Drought and salination are significant factors in land becoming marginal (6% of all land is affected by salt^23^). The interaction between salt or water stress (the latter produced via polyethylene glycol (PEG) treatment) and hormone treatment is a potential route to identify treatments to ameliorate the impacts of stress during germination and seedling establishment. Other factors important for germination include the presence of light. Light was included to determine if there was a change in the optimum conditions for germination in a low light environment^24^. Seed priming has been used extensively and are increasingly common in commercial agriculture^25,26^. Water priming is a simple method which should improve the consistency and reduce the thermal time to germination^27^. We anticipated that factors such as stress, hormones and seed treatments interact in a complex, non-linear fashion to determine the germination and seedling development phenotype. We demonstrate the use of a Taguchi factorial design of experiment to efficiently test the effects and interactions of many potential contributory factors on germination and seedling development in *Miscanthus*.

## Methods and Materials

### Seed sources

The seed were obtained from a synthetic population of five *M. sinensis* plants of Japanese origin crossed in Texas (by CERES Inc., now Land O’Lakes) in 2012. Panicles were harvested and threshed to clean the seed of surrounding material before being stored in a controlled environment seed store at Aberystwyth, UK.

### Germination protocol

For both the range finding tests and the Taguchi experiment a single dish protocol was used. Sixty-four seeds per dish were sterilised with 10% household bleach, rinsed thoroughly, then laid out in a grid, on a 110 × 110 mm square section of steel blue germination paper (Anchor paper co., Minnesota, USA). Beneath the germination paper four layers of blue roll acted as a liquid reservoir. The germination paper was placed in square vented petri dishes, and cultured in a Fitotron 120 Plant Growth Chamber (Loughborough, UK) at 25 °*C* for 11 days.

1 $$GI=\mathop{\sum }\limits_{i\mathrm{=1}}^{n}\,\frac{|({D}_{t}-{D}_{i})\cdot {G}_{i}|}{S}$$

Germination was monitored daily and each seed was scored as germinated if it had produced an extruded radicle of length greater than approximately 1 mm^28,29^. At the end of the experiment the root and epicotyl lengths (mm) of all germinated seedlings were measured. The germination index (GI)^30,31^ was calculated for each dish according to Eq. (1)^30,32^ to provide a single score where *n* is the day of the final counting; *D*_*t*_ the experiment length in days; *D*_*i*_ the number of days until day *i*; *G*_*i*_ the germination count on day *i* and *S* is the total seeds tested^30^. Each seed was followed individually and to each dish was applied one treatment e.g. one concentration of a hormone.

### Range testing

Range finding tests were first used to determine the broad effect of the factors under test and to enable a better selection of factor levels for the subsequent Taguchi experiment. The effects on germination across a wide range of concentrations was determined for abscisic acid (ABA), gibberellic acid (GA), brassinosteroid (BR), auxin (1– naphthaleneacetic acid), NaCl, PEG 4000 and PEG 8000. The range of concentrations tested were chosen from a literature review of available plant studies and were extended in both directions to ensure each experiment captured a wide range of effects. Full details of the ranges tested are in the Supplementary Methods and the Supplementary Results. The four hormones, salt and PEG treatments were diluted in water, with a surfactant (ethanol) if needed, and then added to the germination paper. Two smaller tests were completed with the same seed after commercial priming (primed by Elsoms seeds (Spalding, UK)).

### Factors selected

The factors are the physical and chemical variables in a Taguchi experiment. Each factor can have two or more levels dependent upon the Taguchi design chosen, therefore a treatment is a factor level combination. Each individual treatment can have multiple measurements taken as metrics of the success of the treatment; these can be combined in an Overall Evaluation Criteria (OEC). Germination, growth and establishment of the seedling are important outcomes; therefore, assessments of root and epicotyl growth along with germination were included in the experiments. Because of the interactive effects of hormones, the concentrations in the range-finding test may not necessarily give the same results when in combination with other treatments, so a broad range of concentrations were selected for the experiment.

#### Abscisic acid

**Figure 1:**
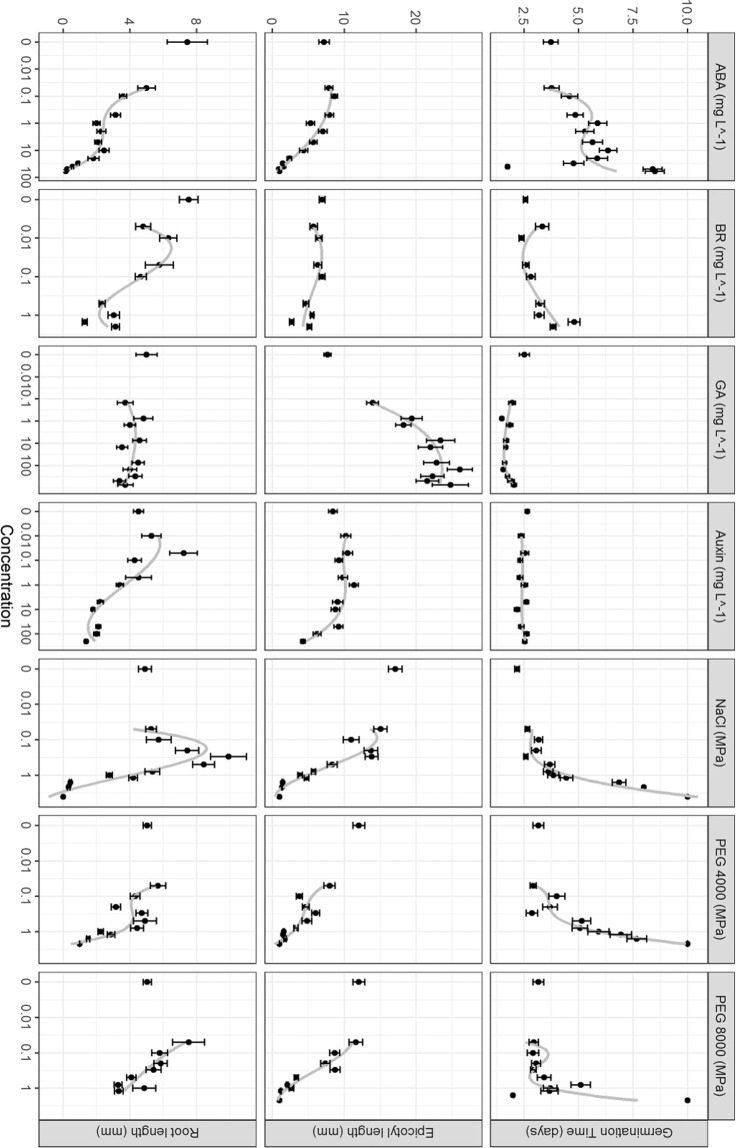
Results from preliminary range tests of the hormone, salt and water stress treatments showing the mean values for each concentration and zero (control) of the treatments with standard error bars. A third order polynomial line (in grey) shows the trend in all concentrations (not including controls).

ABA values of 0.02, 0.2, 2, and 20 mg L^−1^ were chosen to represent the full range in which effects were seen in the range finding (Fig. 1).

#### Gibberellic acid

GA did not have a noticeable secondary effect in the range finding experiment (Fig. 1); therefore, 0.015, 0.15, 1.5, and 15 mg L^−1^ were chosen as representative of the range of effective values.

#### Auxin

Auxin levels used were 0.005, 0.05, 0.5, and 5 mg L^−1^; this cut the long diminishing tail from the range finding experiment short to 5 mg L^−1^ (Fig. 1), and the other levels were selected to stay to a well-distributed exponential scale.

#### Brassinosteroid

BR levels of 0.015, 0.75, 1.5, and 7.5 mg L^−1^ were chosen; 0.75 mg L^−1^ was the concentration that deviated most from the control treatment, 1.5 mg L^−1^ was expected to produce the highest effects, and 7.5 mg L^−1^ used to extend what was seen in the range finding test, which had a small increase in seed performance metrics after 1.5 mg L^−1^ (Fig. 1).

#### Water stress

PEG 8000 and PEG 4000 performed similarly in the range finding tests (Fig. 1 full details in the Supplementary Results). PEG 8000 was chosen because it did not appear to be trapped in the matrix of the germination paper and, due to its size, PEG 8000 is less likely than PEG 4000 to enter the seed.

#### Sodium chloride

NaCl was not further used for water limitation because water limitation by NaCl only affected the seeds at high concentrations (Fig. 1), and the effect of salt toxicity would therefore be difficult to separate from the effects of water limitation.

#### Light

Low light was used instead of complete darkness, because to monitor germination in the dark independent aliquots of seed would be needed for each time point. Light levels were reduced by six layers of bleached white muslin, as a neutral density filter, placed around the dishes. The filter produced a reduction of 73% in photosynthetically active radiation, from 300 to 80 *μ* mol m^−2^ s^−1^, measured using a Skye SKP 215 sensor (Llandrindod Wells, UK). We expected that seedlings would grow larger and healthier under brighter light, possibly with shorter epicotyls and this could be counteracted by the effect of GA on epicotyl length^33^.

#### Priming

Priming was included because of the range test results (Supplementary Results) using primed seed suggested primed seed might have remained in dormancy for too long after being primed and therefore combining primed seed with hormones could demonstrate the activity of hormones in releasing seed from dormancy.

### Taguchi design

A mixed design Taguchi method was used based on the L_16_ (2^3^ 4^4^) table with 16 experimental units, but with four factors at four levels and three factors at two levels (see Table 1). Factors were chosen to go into this design based on their effect on seed outcomes (germination and epicotyl/root elongation) as well as how the multiple factors would fit into one combined method. Each hormone was tested at four levels, PEG 8000 at two levels, light at two levels and primed seed as a two level factor. The four concentrations of the hormones were based on the range finding tests and included a minimum very low concentration, that should have no or near to no effect, a low but active concentration, a main active concentration, and a very high concentration that may have a secondary or inhibitory effects (as shown in the Supplementary Results). The experiment was conducted using the levels shown in (Table 1) and at the limits indicated above. All dishes were prepared and tested at the same time to limit extraneous sources of variation.

**Table 1:** Table of factor levels (very low to very high) for hormone, treatment, or growth condition used in the Taguchi experimental design for each of 16 experimental combinations.

Experi- ment N^o^	ABA (*mgl*^−1^)	GA (*mgl*^−1^)	Water Stress (*Mpa*)	NAA (*mgl*^−1^)	BR (*mgl*^−1^)	Light Level	Primed Seeds
1	V-Low	V-Low	Low	V-Low	V-Low	Low	Yes
2	V-Low	Low	Low	Low	Low	High	No
3	V-Low	High	High	High	High	Low	No
4	V-Low	V-High	High	V-High	V-High	High	Yes
5	Low	V-Low	Low	V-High	High	High	No
6	Low	Low	Low	High	V-High	Low	Yes
7	Low	High	High	Low	V-Low	High	Yes
8	Low	V-High	High	V-Low	Low	Low	No
9	High	V-Low	High	Low	V-High	Low	No
10	High	Low	High	V-Low	High	High	Yes
11	High	High	Low	V-High	Low	Low	Yes
12	High	V-High	Low	High	V-Low	High	No
13	V-High	V-Low	High	High	Low	High	Yes
14	V-High	Low	High	V-High	V-Low	Low	No
15	V-High	High	Low	V-Low	V-High	High	No
16	V-High	V-High	Low	Low	High	Low	Yes

The Taguchi design of experiment (DOE) was produced and primarily analysed using Qualtek-4 (Nutek inc., Michigan, USA) with secondary analysis in R^34^. The Qualtek-4 software calculated the percentage effect of each treatment; this is the primary output of the Taguchi method. This was done by correcting the product of the results for the number of experiments (16) then comparing variations around the mean using the total and per treatment sum of squares^17^. The factors of the sums of squares can then be used to calculate the relative effect of each factor based on the number of levels per factor in this case L_16_ (2^3^ 4^4^). The equations and descriptions for these steps are detailed in Supplementary Methods Table 3.

The Taguchi method allows for multiple responses^15^, e.g. germination and epicotyl length, to be output metrics and this was utilised to analyse several important metrics of germination and early growth. The metrics chosen for the Taguchi analysis were analysed separately to determine what the main effects of each treatment were and if there were any significant interactions between them. The speed of germination as a measure of vigour can be calculated in two ways: First as the mean time to germinate in days, this is the average time taken in a treatment for viable seed to germinate. Secondly 1/T_50_, this is the reciprocal of the time to 50% of viable seed germinated. The second method was used in addition to average time because the 50% point is less easily skewed. GI^30,31^ was also calculated to give a summarial comparison of germination time and percentage (1). Because quick growing seedlings are inherently important for establishment of the crop after germination, epicotyl and root elongation were also assessed along with the epicotyl:root ratio. Data from the fluorescence imaging of the seeds was used to determine photosynthetic activity, both the total area per dish that was photosynthetically active, and the median level of the Fv/Fm reading.

#### Taguchi OEC

2 $$OEC=\left(\frac{{y}_{1}}{{y}_{1max}}\right)\cdot {w}_{1}+\left(\frac{{y}_{2}}{{y}_{2max}}\right)\cdot {w}_{2}+\ldots $$

The Taguchi method utilises a summary statistic, called the Overall Evaluation Criteria (OEC), if the individual responses measured do not generate a consensus as to optimal treatment the OEC can be used [17, p.429]. The OEC can be used to determine which set of treatments were the best for seedlings based on all of the experimental responses, where smaller or larger values for each response are set as optimal (Table 2). To do this, the outputs were normalised then multiplied by subjective weightings given to each of the experimental outputs, based on their importance to the objective of the experiment [17, p.54]. The OEC calculation is shown in Eq. (2) ^35,36^; where *y*_*i*_ is the response measurement, *y*_*i max*_ is the maximum value for the response, and *w*_*i*_ is the weighing of the response. In this study, weighting was done using the principle that the weighting should be higher for the more complex responses that encompass more of the biology of interest. Therefore, the GI was weighted highest at 0.25, because it is an index of speed and quantity of germination, which gives a broad measure of germination success. The epicotyl:root ratio was weighted at 0.2 because it used information from both epicotyl and root elongation, giving an indication of the overall health of the seedling. Next, the total area and median level of Fv/Fm fluorescence were both weighted at 0.15, because these measurements provided an overall assessment of the size and health of the seedlings. The amount of germination and 1/T_50_ of the germination were both weighted at 0.075 because these results represented individual measurements. Lastly the individual measurements of root and epicotyl elongation were both weighted at 0.05 because they represent individual factors, and were considered least important.

**Table 2:** Each experimental setup in the Taguchi experiment ordered by the overall evaluation criteria (OEC) statistic. The combination of factors to achieve the optimal weighted result is at the top of the table.

ABA *mgL*^−1^	GA *mgL*^−1^	Water Stress (*Mpa*)	NAA *mgL*^−1^	BR *mgL*^−1^	Light Level	Primed Seeds	OEC
Low	Low	Low	High	V-High	Low	Yes	84.886
V-Low	Low	Low	Low	Low	High	No	83.049
V-Low	V-Low	Low	V-Low	V-Low	Low	Yes	77.302
High	High	Low	V-High	Low	Low	Yes	74.090
Low	V-High	High	V-Low	Low	Low	No	70.203
Low	V-Low	Low	V-High	High	High	No	68.634
High	V-High	Low	High	V-Low	High	No	66.477
V-Low	High	High	High	High	Low	No	62.291
V-High	V-High	Low	Low	High	Low	Yes	54.014
V-High	High	Low	V-Low	V-High	High	No	46.004
High	V-Low	High	Low	V-High	Low	No	41.693
V-Low	V-High	High	V-High	V-High	High	Yes	33.249
V-High	Low	High	V-High	V-Low	Low	No	21.395
Low	High	High	Low	V-Low	High	Yes	10.220
High	Low	High	V-Low	High	High	Yes	5.296
V-High	V-Low	High	High	Low	High	Yes	4.594

### Taguchi interactions

Interactions between the factors were also reported by the Taguchi analysis. These were given a sensitivity index to characterise the interaction signal against the noise. The interactions with a sensitivity index of over 70% were used to identify possible interactions of factors. Of particular interest were interactions among hormones and the effect of hormones on the response to physical factors.

## Results

### Range testing

The range finding tests consisted of nine experiments. The effects on three of the most basic metrics (germination, epicotyl length and root length) of the four hormones, salt and water stress are shown in Fig. 1. The responses were used to inform the ranges of the factors used in the Taguchi design of experiment (DOE) as detailed in the methods and materials. The other factor tested in the range finding tests was the seed priming, which consisted of two experiments where primed seeds developed smaller seedlings because of a significantly shorter epicotyls in one test and significantly shorter roots, than the control. In contrast, the mean dark-adapted chlorophyll fluorescence response (Fv/Fm) was significantly higher in the primed seed treatment. Full range finding results for all factors are available in the Supplementary Results.

### Taguchi

**Figure 2:**
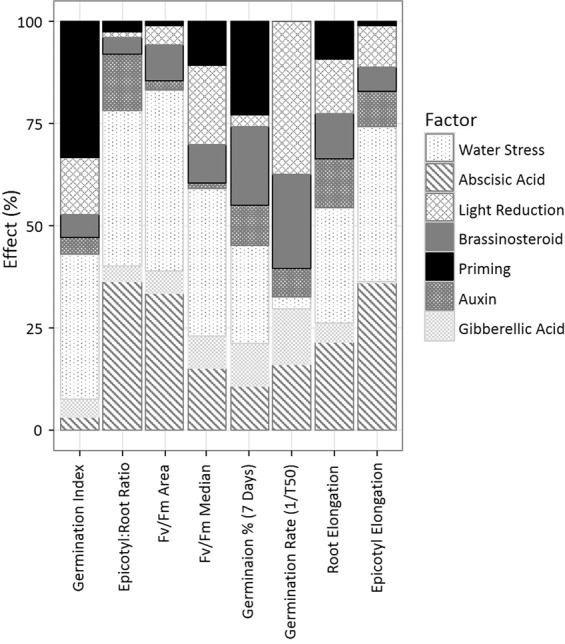
The percentage effect of each factor on each metric of assessment for germination and early seedling growth was calculated from the ANOVA result of the Taguchi.

The results of the Taguchi L_16_ experiment based on all the responses revealed that water stress had the largest effect of any factor this was followed by ABA. Other factors were similar in impact overall but varied in which output they affected most (Fig. 2). The results from the 16 experiments used in the Taguchi analysis are shown in Table 3. Starting with the lowest weighted outputs, epicotyl and root elongations were both similarly shortened by water stress, although epicotyl elongation slightly more so; root was affected by 28% and epicotyl affected by 38%. Epicotyl elongation was also more negativity affected (36%) by ABA than root elongation (21.5%). GA had little effect on either and the effect of GA was particularly small for epicotyl elongation (2.2%), the effect on root elongation was 4.9%. Priming had a limited negative impact on root elongation (9.2%). Next, germination rate as given by 1/T_50_ (to show the speed of germination) was analysed (Fig. 2). This was affected most by levels of light (37.4%), there was a positive effect of low light. Germination rate was also effected by BR (22.9%), which had a more complex set of positive and negative effects than light. There were also effects from GA (13.7%) and ABA (16.1%) treatments. Germination percentage at 7 days differed from germination rate mostly in that there was a much larger effect on germination percentage from priming (22.6% improvement in germination versus a 0.02% decrease in germination rate) and water stress (24.2% and 2.7% negative effect respectively). The reverse was true for light which had the largest effect on germination rate (3% and 37.4% respectively, with the brighter treatment being negative). The combined measure of germination, germination index (GI), was less affected by ABA (3.1%) than was germination percentage (10.9%) or germination rate (16.1%). This was also the case for BR treatment, GI was effected by 5.5% compared to a 19.4% effect on germination percentage and 22.9% effect on germination rate (Fig. 2). Median values of chlorophyll fluorescence described the average photosynthetic activity over each treatment, while total area produced an indication of the total amount of photosynthetically active leaf. The main difference between median and area values was measured in ABA treatments, the area being affected by 33.6% and median by 15.1%. The negative effect of priming was greater for median values of fluorescence (10.8%) than area (0.7%). This was also true for light, the median Fv/Fm was affected by 19.3% whereas total area was affected by 4.9%, both were improved by low light. Total area of photosynthetic activity was also negatively affected by water stress and this effect was more than the median Fv/Fm value, 44.3% to 35.9% respectively (Fig. 2). Lastly, epicotyl:root ratio was most affected and negatively affected by water stress (38.1%), followed closely by ABA (36.3%), auxin was less impactful (13.9%) (Fig. 2).

**Table 3:** Raw data from each of the eight metrics across 16 factorial experiments used in a Taguchi analysis of factors effecting germination and seedling growth. The mean and predicted optimal performance and weightings are shown at the bottom.

Experiment N^o^	Germ- ination Index	Epicotyl:Root	Fv/Fm Area	Fv/Fm Median	Percentage Germ. 7d	Germ. Rate (1/*T*_50_)	Epicotyl Elongation	Root Elongation
1	1.69	3.64	338.6	0.796	29.7	0.031	12.21	3.35
2	3.05	2.52	375.4	0.739	54.7	0.029	8.14	3.24
3	2.34	1.96	133.0	0.776	39.1	0.036	5.87	3.00
4	0.56	2.73	32.4	0.683	12.5	0.019	5.00	1.83
5	2.48	2.69	227.3	0.774	46.9	0.024	6.14	2.29
6	2.34	3.81	345.6	0.797	40.6	0.031	11.08	2.91
7	0.42	1.40	6.2	0.271	7.8	0.027	1.40	1.00
8	2.83	1.79	207.0	0.764	48.4	0.033	6.45	3.61
9	2.28	1.04	10.0	0.68	39.1	0.032	1.36	1.31
10	0.27	1.00	0.5	0.261	4.7	0.028	1.00	1.00
11	2.09	2.94	225.0	0.775	34.4	0.043	9.65	3.28
12	2.59	2.54	161.8	0.694	46.9	0.030	6.50	2.56
13	0.27	1.00	1.0	0.201	4.7	0.031	1.00	1.00
14	1.19	0.88	10.9	0.443	21.9	0.027	1.46	1.67
15	2.17	1.05	60.1	0.735	39.1	0.027	2.85	2.70
16	2.67	1.51	55.7	0.744	46.9	0.031	2.52	1.67
Mean	1.82	2.09	136.9	0.63	34.4	0.030	8.69	2.28
Optimal	4.30	4.64	481.6	1.231	79.6	0.047	13.85	5.03
Weighting	0.25	0.2	0.15	0.15	0.075	0.075	0.05	0.05

**Figure 3:**
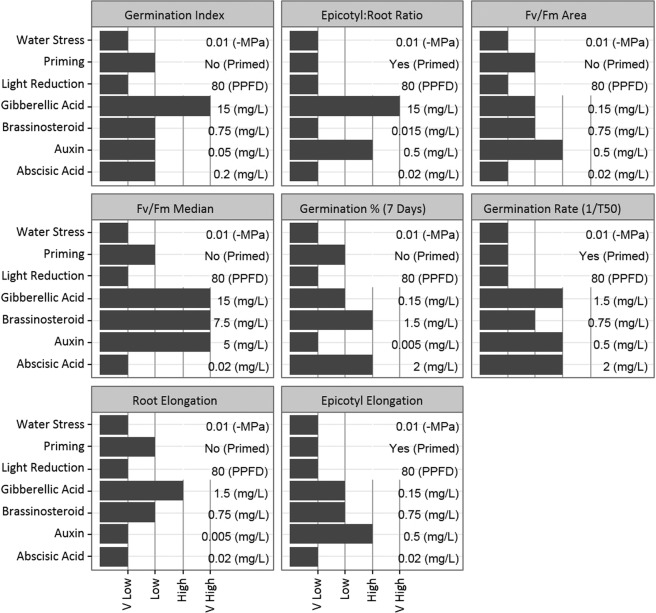
The Taguchi calculated optimum level for each factor for each metric tested. The first three factors have only two levels, here listed with the others as V Low and Low; however, these could be stated as low and high. The optimum level is given next to each (units are abbreviated: *μmolm*^−2^ *s*^−1^ as PPFD and *mgL*^−1^ as *mg*/*L*).

The Taguchi analysis calculated the optimum levels for each factor at each metric (Fig. 3); the usefulness of these values is dependent upon the percentage effects (Fig. 2). For example, the longest epicotyl elongation was in 0.15 *mgL*^−1^ (low) concentration of GA, but the percentage effect of GA on epicotyl elongation was only 2.2% (Fig. 2), so clearly the effect of GA was limited at any level.

#### Single factor effects and interactions

For light, which had only two levels, low light (PAR of 80 *μ* mol m^−2^ *s*^−1^) was an improvement over high light (PAR of 300 *μ* mol m^−1^ s^−1^) in all metrics (Fig. 3). Light had a large overall effect of 13% (Fig. 2). Light was most relevant in speed of germination, followed by percentage germination, having a 2.5% effect on the percentage of seeds germinated (Fig. 2). Light also had a 0.3 mm effect on mean root elongation.

Water stress had a mean effect of 31% on the metrics, with effects over 30% in GI, epicotyl:root, Fv/Fm area and median Fv/Fm, for example water stress resulted in an 86.8 mm^2^ impact on photosynthetic area (Fig. 2). Water stress produced higher measurements at the low level of −0.01 MPa for all metrics (Fig. 3).

The presence of ABA was calculated to have affected the germination percentage by 6.8% (Fig. 2). ABA was optimal for most measurements at the very low treatment level of 20 *mgL*^−1^ (Fig. 3). It had an effect on GI at 200 *mgL*^−1^ but the percentage effect of this was low (3%) (Fig. 2). ABA also had optimal performance at 2 *mgL*^−1^ (Fig. 3) for germination rate (1/T50) and total germination percentage (effect percentages of 16.1 and 10.9%, shown in Fig. 2). There were no points where 20 *mgL*^−1^ was the best treatment; the average best treatment for ABA was 0.54 *mgL*^−1^ (Fig. 3).

GA treatment was never optimal at a near zero (very low) level of 15 *mgL*^−1^ (Fig. 3). The optimum level varied over the 150 *mgL*^−1^ to 15 *mgL*^−1^ range. GA had the biggest effect on germination rate, at 13.7% (Fig. 2), for which the optimal level was calculated as 150 *mgL*^−1^ (low) (Fig. 3). The effect of GA on germination percentage was optimal at 1.5 *mgL*^−1^ (high) (Fig. 3); however, the percentage effect was low at 10.2% (Fig. 2). GA had a low average effect of 6% (Fig. 2).

Auxin (1-naphthaleneacetic acid) was optimal for root elongation and germination percentage at the near zero levels (5 *mgL*^−1^) (Fig. 3). A level of 50 *mgL*^−1^ of auxin was calculated to be optimal for GI, but auxin only had a 4.3% (Fig. 2) effect on GI. Auxin normally performed best at a high level of 0.5 *mgL*^−1^ for epicotyl:root ratio, germination rate, epicotyl elongation, and photosynthetic area (Fig. 3); of these, only the first three demonstrated an effect of more than 5% (Fig. 2). The very high level of auxin (5 *mgL*^−1^) was optimal for median Fv/Fm (Fig. 3), on which it had a low effect (1.2%) (Fig. 2).

Epicotyl:root ratio was the only metric that was optimal at the near zero, very low level of BR (15 *mgL*^−1^) (Fig. 3)), and this was at a low percentage effect (4.2%) (Fig. 2). BR at 0.75 *mgL*^−1^ was optimal for 5 of the 8 metrics (Fig. 3), most significantly germination rate with an effect of 22.9% (Fig. 2). This was followed by root elongation (11.3%) and Fv/Fm area (8.8%). BR was optimal at the high level (1.5 *mgL*^−1^) for germination percentage (Fig. 3); on which it also had a high effect (19.4%) (Fig. 2). The maximum level of BR (7.5 *mgL*^−1^) was only most effective for median Fv/Fm (Fig. 3); on which it had an effect of 9.5% (Fig. 2).

Priming had an average effect of 10%, the largest effect was on GI (33.1%) and germination percentage (22.6%) (Fig. 2). Unprimed seed were optimal (Fig. 3) in all measurements that experienced an effect of more than 10% (Fig. 2). However, primed seed treatment was optimal for epicotyl:root ratio, germination rate, Fv/Fm area, and epicotyl elongation. However, all of these had a low percentage effect, the highest being epicotyl:root ratio at 2.5% (Fig. 2). Overall priming changed germination percentage by 7%.

Unexpectedly GI and germination percentage had no interactions above a sensitivity index of 70%; however, they are related metrics. The first combination with four relevant interactions was GA and BR, which interacted in epicotyl:root ratio, median Fv/Fm, epicotyl elongation, and root elongation. The other pair of factors that interacted at four points was the combination of GA and auxin, which interacted in the measurements of epicotyl:root ratio, Fv/Fm area, epicotyl elongation, and root elongation. Two other pairs of factors interacted. Firstly, water stress and priming interacted in the germination rate, which appeared to show that non-primed seed germinated faster under water stress than primed seed. Secondly, light level and priming interacted in the epicotyl:root ratio, this showed primed seed grew with a larger epicotyl:root ratio under high light than unprimed seed.

#### Effect size and optimisation

The overall evaluation criteria (OEC) calculated weighted combinations of factors (Table 2), this was used in the Taguchi analysis to determine the optimal conditions. The OEC showed an optimal level of 80 *μ*mol m^−2^ s^−1^ for light (low), an optimal Ψ of −0.01 MPa (low), and with the unprimed seed being optimal (Table 4). The optimal hormone levels in the OEC were; 0.02 *mgL*^−1^ for ABA (very low), 0.75 *mgL*^−1^ for BR (low), 0.5 *mgL*^−1^ for auxin (high), and 15 *mgL*^−1^ for GA (very high).

**Table 4:** The percentage effect of each factor in the Taguchi and the level at which it was optimal.

Factor	Percentage Effect OEC	Optimal Level OEC
Abscisic Acid	21.2	V-Low
Gibberellic Acid	1.5	V-High
Auxin	1	High
Brassinosteroid	3.8	Low
Water Stress	50.1	Low
Light	15.2	Low
Priming	7.2	No

The lowest percentage effect was from auxin (1%); the hormones GA and BR, despite being optimal at the higher concentrations, also had low percentage effects (under 4%) on OEC (Table 4). As with other metrics, the strongest interactions in the OEC were GA with auxin and GA with BR, with both over 70% sensitivity. The effect of ABA’s very low optimal level was second only to that of water stress (Table 4). There was also a notable effect of light level (7.2%), indicating low light was favourable for early seedling development.

The Taguchi method can estimate the total optimisation of the factors (Table 2). This shows that the mean epicotyl length of 8.7 mm can be increased to 13.9 mm, and root length from 2.3 to 5 mm. This estimate is over optimistic for measurements with a hard maxima as the Taguchi experiment estimates percentage germination could move from 34.4 to 79.6% when the maximum of any individual treatment was 54.7%, this overestimation is more apparent with the move from mean to optimal Fv/Fm fluorescence (0.63 to 1.23).

## Discussion

The Taguchi method was an efficient method to test the critical factors and interactions between them that achieved high germination and seedling growth, to choose optimal conditions for germination and early seedling development. The statistical method entailed a degree of compromise, because the number of factors and levels was limited to keep a small orthogonal design. Levels of each factor were chosen to cover the full spectrum of effects; however, light and water stress were not tested at as many points as would have been optimal, in order to keep to a small L_16_ design.

The percentage effects of each input gave different results depending on the output. This is as expected because different combinations of treatment should affect seedling germination and growth differently. Epicotyl elongation was slightly more affected by water stress than root elongation; this was because root growth was positively affected by low levels of water stress and negatively affected by high water stress, and epicotyls were only negatively affected. The large effect of seed priming on germination percentage that was not present in germination rate implies that priming has damaged some seed’s ability to germinate but the rate of germination of the population of seed still able to germinate is not affected. This was not the effect expected of this treatment since priming normally begins the germination process which is suspended by drying the seed so that on exposure to water the rate of germination is faster^26,37,38^. This is also seen in water stress where the rate of germination was not affected while the germination percentage was changed. The reverse effect between germination percentage and rate was seen in light treatments; this may be due to the seed germinating more slowly in a low light environment. Therefore, low light germination causes germination speed to be more changeable, but it does not stop seeds germinating. This may be because light is an optional trigger for germination in *Miscanthus* seed.

GI is a metric that includes the combination of the number of seeds germinated each day over seven days. This should mirror the results from the single germination metrics (percentage and rate of germination); however, some factors tested, such as ABA and BR, had a much lower effect on the more complex metric (GI) than the simpler metrics, which may be because factors could have antagonistic effects on the composite metrics thus reducing the overall effect size.

Fv/Fm measured both total photosynthetic area and the median of photosynthetic activity; therefore, differences between the two should indicate the difference between size and health of the plants. The area was much more affected by ABA and water stress, demonstrating these factors affected the size of the seedlings much more than their health. ABA negatively regulates growth, therefore it might be expected that it would affect total photosynthetic area more than median photosynthesis. A similar response was seen in water stress. ABA is highly responsive to water stress and is for example responsible for the regulation of stomatal aperture under drought stress^39,40^ therefore it might be expected that the impacts of water stress and ABA are to some extent similar.

The photosynthetic area was effected by priming and light level by less than 5% while the median Fv/Fm level was affected by more than 10%. This suggests that priming and light impacted photosynthesis more than total area. For light, this is probably due to phototoxic bleaching of leaf tissue^41^. For the priming treatment this is harder to explain, but may suggest priming weakened the seedlings.

Epicotyl to root ratio is of importance as a seedling with a higher ratio may be better able to compete with weeds, but may be less effective in extracting water. This ratio was most affected by water stress, which is understandable because this may force the plant to change the deployment of resources between above and below ground, leading to reduced epicotyl growth. ABA also had a large effect, which is expected because this hormone is known to reduce root growth^42^. Whilst not a large effect, the ratio of root to epicotyl was the measurement most affected by auxin.

It seems counterintuitive but lower light levels had a beneficial effect on all seed measurements; however, this was not zero light as in the preliminary experiments. The high light levels (600 *μ*mol m^−2^ s^−1^) may cause a photo-toxic effect in young seedlings^41^ and could have increased the evaporation rate and led to an interaction with water stress, this may need more investigation to resolve potential confounding interactions.

It is unsurprising that plants with low water stress were shown as more vigorous in all metrics. Water stress had an important effect on metrics particularly the health and growth measurements.

ABA affected the most measurements. As expected, lower concentrations resulted in all metrics indicating seedlings that are more vigorous; the positive effect on GI at 200 *mgL*^−1^ can be discounted due to the low percentage effect (3.1%). However, for germination rate and total germination percentage ABA had the best effect at 2 *mgL*^−1^, suggesting that medium concentrations of ABA may have positively influenced germination. ABA did not interact with GA to effect germination to a high sensitivity index; the absolute level above which the activity of one hormone will dominate is not known for *Miscanthus* and may be one reason for the lack of an expected interaction.

The greatest effect of GA was on rate of germination, for which the high level of GA was best. This could be because GA promotes germination but this effect is saturated and has no further effect at higher concentrations. However, a higher level was optimal for germination percentage indicating that more GA was maintaining a positive effect. There was little to no effect of GA on epicotyl elongation unlike the range finding experiments, epicotyl elongation was controlled largely by the physical factors and ABA. GA did also interact with auxin in four measurements on the seed; this was expected as auxin can counteract GA in the control of dormancy in other seed^43^.

The very low level of auxin produced the longest roots and highest germination percentage which may be explained because auxin acts across a gradient and while it induces growth of root hairs^44^ it may not improve total root elongation. The effect of auxin on high epicotyl to root ratio, germination rate, and epicotyl elongation at a high concentration level could be because moderate to high levels positively affect the epicotyl elongation, and negatively affect the root elongation; thus positively affecting the epicotyl to root ratio and the germination rate. Auxin may have a stimulating effect that increases germination rate but does not increase the total percentage of germination.

Three measurements; root elongation, photosynthetic area, and germination percentage were optimally affected by BR between 0.75 and 1.5 *mgL*^−1^. This suggests that there is an optimal level for stimulating *Miscanthus* seedling germination and growth around 1 *mgL*^−1^. BR did interact with GA to a high level (sensitivity index 83.7%) in many metrics; this supports^43^ who suggested that BR may enhance the effect of GA. BR influenced about 10% of the photosynthetic median, and was optimal in this at its very high treatment level; this suggests that BR can increase the activity of the plant if the plant is oversaturated with BR. This increase in activity may not be good for the plant because the level best associated with most measurements was 10× less concentrated.

The fact that auxin, GA, and BR had notable interactions may have prevented them from contributing a clear percentage effect, rendering their optimal levels less reliable. Further investigation would be required to test each of the interactions identified in the Taguchi and the explanations of the reactions individually; the presented study provides a multidimensional framework as a basis for further study.

The OEC Taguchi analysis showed a method to investigate data to optimise seed germination and early growth of seedlings. Four of the factors studied had more than a 5% effect on the OEC Taguchi. As expected water stress (induced by PEG) was greatest of these which was best when low, ABA was next largest effect and as expected from both the range-finding tests and the literature, ABA was optimal when at its lowest level. The third largest effect was from light, which was optimal when reduced, as it was clearly shown in the individual metrics from the Taguchi analysis. Finally, priming did have a notable and negative effect on the OEC. Our results demonstrate the ability of efficient Taguchi designs to test complex interactions of potentially contributory factors across a number of metrics, if those factors include environmental stresses the Taguchi can examine what treatments may optimise success under that stressed environment. The Taguchi design as a method of screening biologically complex factors refers to every effect and interaction as percentages. This is a realistic way of thinking about the biological systems as every factor applied has some effect, by looking at the effects that produce high percentages appropriate conclusions can be drawn. The Taguchi method produces an OEC to synthesize results from complex outcomes, allowing a broader picture of the success criteria.

In the case of *Miscanthus*, the best scenario applied by the OEC is only useful in ideal conditions and *Miscanthus* is often grown in marginal lands, and seed would need to cope with an increased chance of water stress and salt stress. So by looking at the optimal conditions when fixing water stress factor as ‘high' this reduces the maximum OEC from 85 to 70. This demonstrates that the presence of high water stress with the correct combination of factors does not limit the seedlings as much as the water stress as the biggest factor may imply. When fixing of water stress, it is possible to see that GA treatment becomes much more important. If seedlings could germinate in areas of high water stress, this GA result shows a possible methodology for growing *Miscanthus* seeds in marginal land areas that are water limited or salt contaminated.

## Supplementary information

Supplementary materials.

## Data Availability

The datasets generated and analysed during this study are available in the range finding and testing factors affecting *Miscanthus* germination and early growth data are available in OSF repository, https://osf.io/s5h6f/osf.io/s5h6f.

## References

[1] Meehan PG, Finnan JM, Mc Donnell KP (2013). A Comparison of the Energy Yield at the End User for M. x giganteus Using Two Different Harvesting and Transport Systems. Bioenergy Res..

[2] Hodkinson TR, Renvoize SA (2001). Nomenclature of *Miscanthus* × *giganteus* (*poaceae*). Kew Bull..

[3] Clifton-Brown J (2016). Progress in upscaling *Miscanthus* biomass production for the European bio-economy with seed based hybrids. GCB Bioenergy.

[4] Clifton-Brown John, Harfouche Antoine, Casler Michael D., Dylan Jones Huw, Macalpine William J., Murphy-Bokern Donal, Smart Lawrence B., Adler Anneli, Ashman Chris, Awty-Carroll Danny, Bastien Catherine, Bopper Sebastian, Botnari Vasile, Brancourt-Hulmel Maryse, Chen Zhiyong, Clark Lindsay V., Cosentino Salvatore, Dalton Sue, Davey Chris, Dolstra Oene, Donnison Iain, Flavell Richard, Greef Joerg, Hanley Steve, Hastings Astley, Hertzberg Magnus, Hsu Tsai-Wen, Huang Lin S., Iurato Antonella, Jensen Elaine, Jin Xiaoli, Jørgensen Uffe, Kiesel Andreas, Kim Do-Soon, Liu Jianxiu, McCalmont Jon P., McMahon Bernard G., Mos Michal, Robson Paul, Sacks Erik J., Sandu Anatolii, Scalici Giovanni, Schwarz Kai, Scordia Danilo, Shafiei Reza, Shield Ian, Slavov Gancho, Stanton Brian J., Swaminathan Kankshita, Taylor Gail, Torres Andres F., Trindade Luisa M., Tschaplinski Timothy, Tuskan Gerald A., Yamada Toshihiko, Yeon Yu Chang, Zalesny Ronald S., Zong Junqin, Lewandowski Iris (2018). Breeding progress and preparedness for mass-scale deployment of perennial lignocellulosic biomass crops switchgrass, miscanthus, willow and poplar. GCB Bioenergy.

[5] Deuter, M. Breeding approaches to improvement of yield and quality in Miscanthus grown in Europe. In Lewandowski, I. & Clifton-Brown, J. (eds.) *European Miscanthus Improvement*, 28-52 (Institute of Crop Production and Grassland Research, University of Hohenheim, Stuttgart., University of Hohenheim, Germany, 2000).

[6] Ashman Chris, Awty-Carroll Danny, Mos Michal, Robson Paul, Clifton-Brown John (2018). Assessing seed priming, sowing date, and mulch film to improve the germination and survival of direct-sownMiscanthus sinensisin the United Kingdom. GCB Bioenergy.

[7] Valentine J (2012). Food vs. fuel: the use of land for lignocellulosic ‘next generation’ energy crops that minimize competition with primary food production. GCB Bioenergy.

[8] Kalinina O (2017). Extending Miscanthus Cultivation with Novel Germplasm at Six Contrasting Sites. Front. Plant Sci..

[9] Gallagher, E. *et al*. The Gallagher Review of the indirect effects of biofuels production. Tech. Rep., Renewable Fuels Agency (2008).

[10] Clifton-Brown JC, Schwarz KU, Hastings AFSJ (2015). History of the development of Miscanthus as a bioenergy crop: From small beginnings to potential realisation. Biol. Environ..

[11] Zhuang D, Jiang D, Liu L, Huang Y (2011). Assessment of bioenergy potential on marginal land in China. Renew. Sustain. Energy Rev..

[12] Gray C. T. (1988). Introduction to quality engineering: Designing quality into products and processes, G. Taguchi, Asian productivity organization, 1986. number of pages: 191. price: $29 (U.K.). Quality and Reliability Engineering International.

[13] Yaldagard M, Mortazavi SA, Tabatabaie F (2008). Application of Ultrasonic Waves as a Priming Technique for Accelerating and Enhancing the Germination of Barley Seed: Optimization of Method by the Taguchi Approach. J. Inst. Brew..

[14] Tong L-I, Su C-T, Wang C-H (1997). The optimization of multi-response problems in the Taguchi method. Int. J. Qual. & Reliab. Manag..

[15] Rao RS, Kumar CG, Prakasham RS, Hobbs PJ (2008). The Taguchi methodology as a statistical tool for biotechnological applications: A critical appraisal. Biotechnol. J..

[16] Pourjavadi A, Salimi H, Amini-Fazl MS, Kurdtabar M, Amini-Fazl AR (2006). Optimization of synthetic conditions of a novel collagen-based superabsorbent hydrogel by Taguchi method and investigation of its metal ions adsorption. J. Appl. Polym. Sci..

[17] Roy, R. K. *Design of experiments using the Taguchi approach: 16 steps to product and process improvement* (John Wiley & Sons, 2001).

[18] Steber CM, McCourt P (2001). A role for brassinosteroids in germination in Arabidopsis. Plant Physiol..

[19] Belin C, Megies C, Hauserová E, Lopez-Molina L (2009). Abscisic acid represses growth of the Arabidopsis embryonic axis after germination by enhancing auxin signaling. The Plant Cell.

[20] Christian EJ, Goggi AS, Moore KJ (2014). Temperature and Light Requirements for Miscanthus sinensis Laboratory Germination Test. Crop. Sci..

[21] Aso T (1976). Studies on the germination of seeds of Miscanthus sinensis Anderss. Sci. reports Yokohama Natl. Univ. Sect. II, Biol. geological sciences.

[22] Beringer T, Lucht W, Schaphoff S (2011). Bioenergy production potential of global biomass plantations under environmental and agricultural constraints. GCB Bioenergy.

[23] Zhang H (2010). The effects of salinity and osmotic stress on barley germination rate: sodium as an osmotic regulator. Annals Bot..

[24] Ellis RH, Hong TD, Roberts EH (1989). Response of Seed Germination in Three Genera of Compositae to White Light of Varying Photon Flux Density and Photoperiod. J. Exp. Bot..

[25] Sathish S, Sundareswaran S, Ganesan N (2011). Influence of Seed Priming on Physiological Performance of Fresh and Aged Seeds of Maize Hybrid [COH (M) 5] and it’s Parental Lines. ARPN J. Agric. Biol. Sci..

[26] Sharma AD, Rathore SVS, Srinivasan K, Tyagi RK (2014). Comparison of various seed priming methods for seed germination, seedling vigour and fruit yield in okra (Abelmoschus esculentus L. Moench). Sci. Hortic..

[27] Ellis RH, Butcher PD (1988). The Effects of Priming and’Natural’ Differences in quality amongst Onion seed lots on the responce of the rate of germination to temperature and the Identification of the charicteristics under genotypic control. J. Exp. Bot..

[28] Bewley JD (1997). Seed germination and dormancy. The Plant Cell.

[29] Ellis, R. H., Hong, T. D. & Roberts, E. H. *Handbook of seed technology for genebanks*. *Volume I*. *Principles and methodology* (1985).

[30] Ranal MA, de Santana DG (2006). How and why to measure the germination process?. Braz. J. Bot..

[31] Walker-Simmons M (1987). ABA Levels and Sensitivity in Developing Wheat Embryos of Sprouting Resistant and Susceptible Cultivars. Plant Physiol..

[32] Melville, A. H., Galletta, G. J., Draper, A. D. & Ng, T. J. Seed germination and early seedling vigor in progenies of inbred strawberry selections. *HortScience* (1980).

[33] Lockhart JA (1956). Reversal of the light inhibition of pea stem growth by the gibberellins. Biol..

[34] R Core Team. R: A Language and Environment for Statistical Computing, http://www.r-project.org/ (2015).

[35] Roy, R. K. *A primer on the Taguchi method* (Society of Manufacturing Engineers, Dearborn, United States, 2010), 2nd revise edn.

[36] Subba Rao C, Madhavendra SS, Sreenivas Rao R, Hobbs PJ, Prakasham RS (2008). Studies on improving the immobilized bead reusability and alkaline protease production by isolated immobilized bacillus circulans (MTCC 6811) using overall evaluation criteria. Appl. Biochem. Biotechnol..

[37] Giri, G. S. & Schillinger, W. F. Seed Priming Winter Wheat for Germination, Emergence, and Yield. *Crop. Sci*. **43** (2003).

[38] Hussian, I. *et al*. Seed priming: a tool to invigorate the seeds. *Sci. Agric*. **3**, http://www.pscipub.com/Journals/Data/JList/ScientiaAgriculturae/2014/Volume3/Issue3/2.pdf, 10.15192/PSCP.SA.2014.3.3.122128 (2014).

[39] Bunce J (1990). Abscisic acid mimics effects of dehydration on area expansion and photosynthetic partitioning in young soybean leaves. Plant, Cell & Environ..

[40] Stoll M, Loveys B, Dry P (2000). Hormonal changes induced by partial rootzone drying of irrigated grapevine. J. experimental botany.

[41] Maizel A, Von Wangenheim D, Federici F, Haseloff J, Stelzer EHK (2011). High-resolution live imaging of plant growth in near physiological bright conditions using light sheet fluorescence microscopy. Plant J..

[42] Wang L (2011). Auxin response Factor2 (ARF2) and its regulated homeodomain gene HB33 mediate abscisic acid response in Arabidopsis. PLoS Genet..

[43] Shu Kai, Liu Xiao-dong, Xie Qi, He Zu-hua (2016). Two Faces of One Seed: Hormonal Regulation of Dormancy and Germination. Molecular Plant.

[44] Pitts RJ, Cernac A, Estelle M (1998). Auxin and ethylene promote root hair elongation in Arabidopsis. The Plant J..

