# Supplementary material for: Using a Taguchi DOE to investigate factors and interactions affecting germination in Miscanthus sinensis
Source: Sci Rep. 2020 Jan 31;10:1602. doi: 10.1038/s41598-020-58322-x (PMC6994594; doi:10.1038/s41598-020-58322-x)
Supplement: Supplementary file 1 — Supplementary materials. [file 41598_2020_58322_MOESM1_ESM.pdf]

Using a Taguchi DOE to investigate factors and interactions affecting germination in *Miscanthus sinensis*

Danny Awty-Carroll[1,*], Sreenivas Ravella[1], John Clifton-Brown[1], and Paul Robson[1]

[1]Institute of Biological, Environmental and Rural Sciences, Gogerddan, University of Aberystwyth, SY23 3EE, UK
[*]dga1@aber.ac.uk

Supplementary information on range finding tests

Methods and materials

Abscisic acid

The range test for abscisic acid (ABA) used concentrations of 0, 0.05, 0.1, 0.5, 1, 2, 5, 10, 20, 30, 40, 50, and 60 mg L^{-1}. The concentration range was based on[1] who used 0.2 to 1 μM of ABA in a study of germination in *Arabidopsis*. Similarly,[2] used 0.5 to 3 μM; therefore, a complete range would be from 0.2 to 3 μM (0.05 to 0.8 mg L^{-1}). This was extended upwards to include any secondary effects that may occur at higher concentrations.

Gibberellic acid

The range test for Gibberellic acid (GA) used concentrations of 0, 0.15, 0.75, 1.5, 7.5, 15, 75, 150, 300, 500, and 750 mg L^{-1}. This was based on[3] who tested 10 to 100 mg L^{-1} of GA on *Miscanthus* and found all concentrations had the same effect. Also[4] used 100 mg L^{-1} on rice, while testing on *M. sinensis* seed has been as high as 500 mg L^{-1}[5]. The concentration range was extended in both directions to ensure the experiment captured a wide range of effects.

Brassinosteroid

Epibrassinolide was used as a brassinosteroid (BR) because it has previously been used in germination[2,6], root growth[7], and hormone regulation[8] studies. Concentrations of 0, 0.001, 0.005, 0.01, 0.05, 0.1, 0.5, 1, 1.5, and 2 mg L^{-1} were used. This range was based on[7] who used a range from 0.1 to 10 nM of BR on *Arabidopsis* roots,[6] who used 0.1 to 10 μM of BR and NaCl which affected cucumber germination, and[2] who used 0.5 to 2.5 μM of BR in *Arabidopsis* germination. This range could be restricted because[6] only saw a noticeable negative effect on the wild type starting at 1 nM; giving a range of 1 nM to 10 μM (0.005 to 4.8 mg L^{-1}) of BR. However, due to the cost of epibrassinolide and the work by[2,7], the upper limit was reduced to 2 mg L^{-1} (4.2 μM).

Auxin

The auxin used was 1–naphthaleneacetic acid (NAA), due to it being frequently used for germination and seedling root growth studies[1,7,9–11]. 1 mL of ethanol was used (0.3 g L^{-1}) to aid solubilising the hormone. The upper range was based on[10] who used 100 to 500 nM of NAA which equates to approximately 0.02 to 0.1 mg L^{-1}.[7] used lower concentrations of 0.1 to 10 nM but observed the same inhibition of root growth.[1] used an upper concentration of 27 μM ($\approx$ 5 mg L^{-1}) when working with ginseng roots. The spread of the previously used concentrations was extended and the following range of NAA was tested with Miscanthus seed: 0, 0.01, 0.05, 0.1, 0.5, 1, 5, 10, 50, 100, and 200 mg L^{-1}.

Sodium chloride

An experiment using NaCl to reduce water availability and test the impact of salt toxicity was included, the levels of salt used were based a wide range of possible desired water potentials (Ψ). These were calculated using a derivation of the Hoff equation[12] equation (1); where Ψ is water potential, i is the number of ions formed, M is the molarity, R is the pressure constant, and T is the temperature in °K. Seed were put into 12 Petri dishes with of 0, -0.05, -0.1, -0.2, -0.3, -0.5, -0.8, -1, -1.2, -1.6, -2.2 and -4.1 MPa. This range was used because[13] used -0.5 to -2.5 MPa rage of NaCl in barley and germination did not cease within this range. Other germination studies used similar or lower ranges of NaCl[14–16].

$$\Psi = iMRT \tag{1}$$

$$\Psi = \alpha \cdot C + \beta \cdot C^2 \quad (2)$$

Water stress (PEG)

The PEG range finding experiment was designed to align with the NaCl experiment in terms of water potential (Ψ). PEG avoided any effect of ion toxicity associated with NaCl; however, other problems are possible for example, large PEG molecule could hypothetically become immobilised within the blue germination paper and there is some evidence that low molecular mass PEG molecules could penetrate a plant or seed and cause a toxicity[17,18]. As such it is unclear if high or low Molecular weight PEG would be preferable and so both PEG 4000 and 8000 were tested. To compare and align osmotic pressures different equations were requried for NaCl and PEG. NaCl obeys the Hoff Equation (1) but PEG which is a large coiled molecule binds water according to the Tyndall effect[19,20].[21] used an equation (2) to model the osmotic potentials of different non-ideal solutions. α and β are listed for a variety of PEG molecular masses, from these a variety of curves can be calculated of the water potential in solutions of PEG. The water potentials using PEG 8,000 and 4,000 were calculated using the best suited equation. The general PEG 8,000 equation was used (3) from[22]; where T is temperature in degrees Celsius and Π is the water potential in bar. The[22] equation (3) would be best for calculating a very wide range of PEG solutions; however, the PEG 8,000 specific calculation (2)[21] was used because there is a large difference between PEG 6,000 and PEG 10,000[22].

$$[PEG] = \frac{4 - (5.16\Psi T - 560\Psi + 16)^{0.5}}{2.58T - 280} \quad (3)$$

Priming

Seed was water primed by imbibing until the seed were close to germination then drying back to the original moisture content. The priming process was performed by Elsoms Seeds (Spalding, UK), the same seed are used in[23] The effects of priming on germination and early growth of *Miscanthus* seed were tested in two experiments. Primed and unprimed *M. sinensis* seed were germinated on two plates, 64 seed of each, and measurements taken daily. Germination data were analysed using a Kruskal-Wallis rank sum or a t-test depending on the normality of the data. A second replicated experiment into priming used three dishes separated into two sections: 50 primed and 50 unprimed *M. sinensis* seed were randomly assigned to left or right side. The seeds were grown for 37 days to allow more difference in measurements of epicotyl and root growth. Fluorescence images for each plate were gathered through-out the experiment and at the end of the experiment using the CF Imager (Technologica Ltd, Colchester UK). Water was added as necessary, to keep the blue germination paper fully wet. The mean fluorescence Fv/Fm values and mean total fluorescence areas for each side of the dish were analysed with a paired t-test or a Wilcoxon signed rank when lacking a normal distribution. Epicotyl and root elongation was analysed via t-tests or Kruskal-Wallis rank sums depending on normality. A Kruskal-Wallis was also used to determine if there was a significant difference in the total germination counts at the end of the experiment.

Results

Abscisic acid

The final mean elongation of the epicotyls exposed to concentrations of GA that were more than or equal to 0.75 mg L^{-1} was 22.1 mm, which is longer than the control at 7.7 mm (Fig 1). Root elongations did not appear to be affected by the GA concentration. The time taken for the individual seeds to germinate under the influence of GA showed no clear pattern. GA concentration produced maximum germination between 1 and 10 mg L^{-1} This is most noticeable for GI, which was 4.09 in the control, rising to 4.65 at 7.5 mg L^{-1} and between 3.3 and 4.4 in the remaining treatments.

Gibberellic acid

There was a negative effect of ABA on root and epicotyl elongation (Fig 1), particularly at higher concentrations (> 20 mg L^{-1}). However, this is only a difference between a median of 5 mm for the control and 7 mm for concentrations less than or equal to 5 mg L^{-1}. The mean differences for both is around 7.19 mm. Time to 50% germination was 4 days for the control and for 0.05 mg L^{-1}, before increasing to 8 days at 0.1 mg L^{-1} after which 50% of seeds do not germinate. GI dropped from over 4 in the first two concentrations to 3.6 at 0.1 mg L^{-1} then 2.5 at 0.5 mg L^{-1}. It then stabilised around 2 mg L^{-1} until more than 30 mg L^{-1} after which it remained less than 1.

Brassinosteroid

Brassinosteroid (BR) had no effect on epicotyl elongation and only a minor effect on root elongation (Fig 1), which decreased at concentrations above 0.5 mg L^{-1}, and both epicotyl and root elongation fluctuated more above 0.1 mg L^{-1}. The change in mean root elongation occurred from a mean of 6.6 mm at 0 mg L^{-1} to a mean of 2.5 mm as an average of the highest three concentrations (1, 1.5 & 2 mg L^{-1}). Epicotyl elongation went from 7.4 mm to 4.5 mm over the same range of concentrations. The median time taken for seeds to germinate remained at two days at concentrations below 0.5 mg L^{-1}. At concentrations of 0.5 or 1 mg L^{-1}, the median time to germinate was three days and four days at higher concentrations. Only 22% of seed germinate at 1.5 mg L^{-1}; however, this result appears to be an outlier, because across all other dishes germination was between 41% and 53% regardless of BR concentration.

Auxin

Auxin (NAA) appeared to have a positive effect on epicotyl and root elongation at 0.01 to 0.05 mg L^{-1}, after which the root elongation was less than the control (Fig 1). Epicotyl elongations was unaffected or greater until over 50 mg L^{-1} auxin. It was observed during the test that the roots appeared fluffier, probably due to more root hairs being visible in the auxin dishes. Auxin had little noticeable effect on the speed of germination. The mean number of days for a seed to germinate stayed at around 2.5 days regardless of concentration. However there was a small increase in median germination speed from 2.5 to 2 days comparing the control treatment to all concentrations of auxin.

Sodium chloride

Germination time increased below -0.1 MPa (20 mM) (Fig 1). This was a small effect and larger changes in germination time were not seen until water potential reached less than -1 MPa (201 mM). GI showed similar patten as the proportion of seed germinated and at the end of the test dropped approximately 12.8% per MPa. Little germination was recorded in the higher salt concentrations, at -2.2 and -4.1 MPa (444-827 mM). Epicotyl elongation decreased linearly with the nonlinear x-axis (Ψ). At water potentials lower than -0.8 MPa (161 mM) the increase in salt had a positive effect on root elongation, as the pressure increased the effect was negative similar to the effect of salt on epicotyls.

Water stress (PEG)

Epicotyl elongations and to a lesser extent root elongation declined with water potential (Ψ) in PEG treatments (Fig 1). The decline began at 0.05 MPa in both epicotyl and root but decreased more rapidly after a Ψ of 0.3 MPa for epicotyl elongation and after a Ψ of 1.2 MPa for root elongation. In both cases, seeds were more affected by the PEG 8000 than the PEG 4000, as seen in the more consistent drop for PEG 8000. There was a sharper decrease of seedling epicotyl elongation in PEG 8000. Time taken for seeds to germinate rose sharply around -0.5 MPa for both PEG 4000 and 8000. Seed in PEG 4000 germinated more slowly and over an inter-quartile range of 2 to 6 days, compared to PEG 8000, where germination occurred over an inter-quartile range of 2 to 3 days. Seeds at levels of -1.6 and -2.2 MPa had a germination proportion of $\sim$0.1 in PEG 4000 and $\sim$0 in PEG 8000.

Priming

Epicotyl elongation was the only significant response to priming in the first priming test; control seed produced longer epicotyls at 17.1 mm compared to 13.3 mm from primed seed. There was no significant difference in germination percentage or epicotyl

length in the second priming experiment but the priming treatment resulted in seedlings with a significantly shorter mean root length according to a comparison of means using Student's t-test. The mean dark-adapted chlorophyll fluorescence response (Fv/Fm) was significantly higher in primed seed when tested using a Wilcoxon signed rank ($P < 0.01$). However, the mean total fluorescence area was significantly higher in unprimed seedlings ($P < 0.01$).

Supplementary table 1: Taguchi percentage effects

Table of percentage effects of each factor on each metric from the Taguchi analysis. Metric effects may not total to 100% due to rounding.

**Factor**	**Germination Index**	**Epicotyl :Root**	**Fv/Fm Area**	**Fv/Fm Median**	**Percentage Germ 7d**	**Germ Rate** $(1/T_{50})$	**Epicotyl Elongation**	**Root Elongation**
Abscisic Acid	3.1	36.3	33.6	15.1	10.9	16.1	36.1	21.5
Gibberellic Acid	4.4	3.9	5.4	8.1	10.2	13.7	0.2	4.9
Auxin	4.3	13.9	2.3	1.2	9.6	7.2	8.7	11.9
Brassinosteroid	5.5	4.2	8.8	9.5	19.4	22.9	5.8	11.3
Water Stress	35.6	38.1	44.3	35.9	24.2	2.7	38.0	28.0
Low Light	14.1	1.0	4.9	19.3	3.0	37.4	10.4	13.1
Priming	33.1	2.5	0.7	10.8	22.6	0.0	0.8	9.2

Supplementary table 2: Taguchi dosage effects

The optimal dosage level for each factor on each metric recorded.

Factor	Germ- ination Index	Epicotyl :Root	Fv/Fm Area	Fv/Fm Median	Percentage Germ 7d	Germ Rate $(1/T_{50})$	Epicotyl Elongation	Root Elongation	Units
Abscisic Acid	0.2	0.02	0.02	0.02	2	2	0.02	0.02	$mg L^{-1}$
Gibberellic Acid	15	15	0.15	15	0.15	1.5	0.15	1.5	$mg L^{-1}$
Auxin	0.05	0.5	0.5	5	0.005	0.5	0.5	0.005	$mg L^{-1}$
Brassinosteroid	0.75	0.015	0.75	7.5	1.5	0.75	0.75	0.75	$mg L^{-1}$
Low Light	80	80	80	80	80	80	80	80	$PPFD$
Water Stress	0.01	0.01	0.01	0.01	0.01	0.01	0.01	0.01	$-MPa$
Priming	no	yes	no	no	no	yes	yes	no	

Supplementary table 3: Taguchi equations

The important calculations included in the Taguchi ANOVA.

**Quantity**	**Notation**	**Calculation**	**Description**
Number of Experiments	N		
Correction Factor	C.F.	$\frac{T^2}{N}$	Total value of experimental data squared divided by the number of experiments conducted
Total Sum of Squares	S'_T	$\sum_{i=1}^{9}(Y^2{}_i) - C.F.$	The sum of squares measures each individual value's deviation from the mean value of the dataset
Variance	V_F	$\frac{S_F}{f_F}$	Variance is used to represent distribution of data about the mean value of a dataset
F-Ratio	F_A	$\frac{V_F}{V_e}$	Variance resulting from the effects of a factor and that from the error term
Factor Sum of Squares	S_F	$\frac{A_1^2}{N_{A1}} + \frac{A_2^2}{N_{A2}} - C.F.$	The factor sum of squares measures individual value's deviation from the mean value of the dataset produced by a specific factor, e.g. salts or minerals
The variance of the Error term	V_e	$\frac{S_{Error}}{df_F}$	The sum of the observed values deviance from the 'true' value
Pure Sum of Squares	S'_F	$S_F - (V_e \times f_F)$	
Percentage Influence	P_F	$\frac{S'_F}{S_T}$	The percentage influence of a given factor on the overall variance exhibited by the experimental dataset
Degrees of Freedom	df	N-1	Degrees of Freedom (df) quantifies the volume of information that can be uniquely determined from a particular dataset

References

1. Belin, C., Megies, C., Hauserová, E. & Lopez-Molina, L. Abscisic acid represses growth of the Arabidopsis embryonic axis after germination by enhancing auxin signaling. *The Plant Cell* **21**, 2253–68, DOI: 10.1105/tpc.109.067702 (2009).
2. Steber, C. M. & McCourt, P. A role for brassinosteroids in germination in Arabidopsis. *Plant Physiol.* **125**, 763–769, DOI: 10.1104/pp.125.2.763 (2001).
3. Aso, T. Studies on the germination of seeds of Miscanthus sinensis Anderss. *Sci. reports Yokohama Natl. Univ. Sect. II, Biol. geological sciences* **23**, 27–37 (1976).
4. Dong, Y. *et al.* Mapping of quantitative trait loci for gibberellic acid response at rice (Oryza sativa L.) seedling stage. *Plant Sci.* **170**, 12–17, DOI: 10.1016/j.plantsci.2005.07.021 (2006).
5. Christian, E. J., Goggi, A. S. & Moore, K. J. Temperature and Light Requirements for Miscanthus sinensis Laboratory Germination Test. *Crop. Sci.* **54**, 1–5, DOI: 10.2135/cropsci2013.03.0187 (2014).
6. Wang, B., Zhang, J., Xia, X. & Zhang, W.-H. Ameliorative effect of brassinosteroid and ethylene on germination of cucumber seeds in the presence of sodium chloride. *Plant Growth Regul.* **65**, 407–413, DOI: 10.1007/s10725-011-9595-9 (2011).
7. Müssig, C., Shin, G.-H. & Altmann, T. Brassinosteroids promote root growth in Arabidopsis. *Plant Physiol.* **133**, 1261–1271, DOI: 10.1104/pp.103.028662 (2003).
8. Unterholzner, S. J. *et al.* Brassinosteroids Are Master Regulators of Gibberellin Biosynthesis in Arabidopsis. *The Plant Cell* **27**, 1–13, DOI: 10.1105/tpc.15.00433 (2015).
9. Rahman, A. Auxin is a Positive Regulator for Ethylene-Mediated Response in the Growth of Arabidopsis Roots. *Plant Cell Physiol.* **42**, 301–307, DOI: 10.1093/pcp/pce035 (2001).
10. Rosquete, M. R. *et al.* An auxin transport mechanism restricts positive orthogravitropism in lateral roots. *Curr. Biol.* **23**, 817–822, DOI: 10.1016/j.cub.2013.03.064 (2013).
11. Wang, L. *et al.* Auxin response Factor2 (ARF2) and its regulated homeodomain gene HB33 mediate abscisic acid response in Arabidopsis. *PLoS Genet.* **7**, e1002172, DOI: 10.1371/journal.pgen.1002172 (2011).
12. Lewis, G. N. The Osmotic Pressure of Concentrated Solutions, and the Laws of the Perfect Solution. *J. Am. Chem. Soc.* **34**, 668–683, DOI: 10.1021/ja01947a002 (1908).
13. Zhang, H. *et al.* The effects of salinity and osmotic stress on barley germination rate: sodium as an osmotic regulator. *Annals Bot.* **106**, 1027–35, DOI: 10.1093/aob/mcq204 (2010).
14. Dodd, G. L. & Donovan, L. A. Water Potential and Ionic Effects on Germination and Seedling Growth of Two Cold Desert Shrubs. *Am. J. Bot.* **86**, 1146–1153 (1999).
15. Gummerson, R. J. The Effect of Constant Temperatures and Osmotic Potentials on the Germination of Sugar Beet. *J. Exp. Bot.* **37**, 729–741 (1986).
16. Koger, C. H., Reddy, K. N. & Poston, D. H. Factors affecting seed germination, seedling emergence, and survival of texasweed (Caperonia palustris). *Weed Sci.* **52**, 989–995, DOI: 10.1614/WS-03-139R2 (2004).
17. Lagerwerff, J. V., Ogata, G. & Eagle, H. E. Control of Osmotic Pressure of Culture Solutions with Polyethylene Glycol. *Science* **133**, 1486–7, DOI: 10.1126/science.133.3463.1486 (1961).
18. Lawlor, D. W. Absorption of Polyethylene Glycols by Plants and their Effects on Plant Growth. *New Phytol.* **69**, 501–513, DOI: 10.1111/j.1469-8137.1970.tb02446.x (1970).
19. Mcclendon, J. H. The osmotic pressure of concentrated solutions of polyethylene glycol 6000, and its variation with temperature. *J. Exp. Bot.* **32**, 861–866, DOI: 10.1093/jxb/32.4.861 (1981).
20. Steuter, A. A., Mozafar, A. & Goodin, J. O. E. R. Water potential of aqueous polyethylene glycol. *Plant Physiol.* **67**, 64–67, DOI: 10.1104/pp.67.1.64 (1981).
21. Money, N. P. Osmotic Pressure of Aqueous Polyethylene Glycols : Relationship between Molecular Weight and Vapor Pressure Deficit. *Plant Physiol.* **91**, 766–769, DOI: 10.1104/pp.91.2.766 (1989).
22. Michel, B. E. Evaluation of the water potentials of solutions of polyethylene glycol 8000 both in the absence and presence of other solutes. *Plant Physiol.* **72**, 66–70, DOI: 10.1104/pp.72.1.66 (1983).
23. Ashman, C., Awty-Carroll, D., Mos, M., Robson, P. & Clifton-Brown, J. Assessing seed priming, sowing date, and mulch film to improve the germination and survival of direct-sown Miscanthus sinensis in the United Kingdom. *GCB Bioenergy* DOI: 10.1111/gcbb.12518 (2018).
